# The oligosaccharides 6’-sialyllactose, 2’-fucosyllactose or galactooligosaccharides do not directly modulate human dendritic cell differentiation or maturation

**DOI:** 10.1371/journal.pone.0200356

**Published:** 2018-07-10

**Authors:** Olaf Perdijk, R. J. Joost van Neerven, Erik van den Brink, Huub F. J. Savelkoul, Sylvia Brugman

**Affiliations:** 1 Cell Biology and Immunology group, Wageningen University, Wageningen, the Netherlands; 2 FrieslandCampina, Amersfoort, the Netherlands; INSERM, FRANCE

## Abstract

Breast milk plays an important role in immune development in early life and protects against diseases later in life. A wide range of the beneficial effects of breast milk are attributed to human milk oligosaccharides (HMOs) as well as components such as vitamin D3 (VitD3) or TGFβ. One mechanism by which HMOs might contribute to immune homeostasis and protection against disease is the induction of a local tolerogenic milieu. In this study we investigated the effect of the HMOs 6’-sialyllactose (6’SL) and 2’-fucosyllactose (2’FL) as well as prebiotic galactooligosaccharides (GOS) on DC differentiation and maturation. Isolated CD14+ monocytes were cultured for six days in the presence of GM-CSF and IL-4 with or without 6’SL, 2’FL, GOS, VitD3 or TGFβ. Additionally, immature VitD3DC, TGFβDC and moDC were used as different DC types to investigate the effect of 6’SL, 2’FL and GOS on DC maturation. Surface marker expression and cytokine production was measured by flow cytometry and cytometric bead array, respectively. Unlike TGFβ and vitD3, the oligosaccharides 6’SL, 2’FL and GOS did not influence DC differentiation. Next, we studied the effect of 6’SL, 2’FL and GOS on maturation of moDC, VitD3DC and TGFβDC that showed different profiles of HMO-binding receptors. 6’SL, 2’FL and GOS did not modulate LPS-induced maturation, even though their putative receptors were present on the different DCs types. Thus, whereas VitD3 and TGFβ halt DC differentiation, which results in phenotypically distinct tolerogenic DCs, 6’SL, 2’FL and GOS do not alter DC differentiation or maturation of *in vitro* differentiated DC types.

## Introduction

Our mucosal surfaces are continuously exposed to foreign antigens that can be harmless or harmful. The mucosal immune system needs to distinguish between these antigens to mount regulatory or inflammatory responses. Proper development of the mucosal immune system in early life is therefore essential for health later in life. Breast milk plays an important role in immune development in early life [1]. It has become clear that breastfeeding is associated with lower child morbidity, higher cognitive abilities and reductions in overweight and diabetes [2]. Nevertheless, only a third of the infants are exclusively breastfed in the first 6 months of life worldwide [2]. Thus, the majority of the infants is currently depending on infant formula. Studying the functionality of breast milk components individually may identify components that aid in mucosal immune development and may improve infant formula.

Breast milk contains proteins, lactose, a wide variety of oligosaccharides, fatty acids and vitamins. These breast milk components contribute to the development of the mucosal immune system and maintain homeostasis in the neonate [3]. Moreover, breast milk components enhance epithelial gut maturation, reduce viral infections, influence the colonisation of the GI tract and promote the development of tolerance [3]. A wide range of these beneficial effects are attributed to human milk oligosaccharides (HMOs) [4]. HMOs escape hydrolysis in the intestine and are metabolised by the microbiota in the colon—promoting the outgrowth of bacteria. Additionally, a small fraction of HMOs is taken up into the circulation [5–7]. HMOs can be subdivided into acidic and neutral HMOs. Acidic HMOs contain sialic acid, and neutral HMOs can be subdivided into fucosylated or non-fucosylated HMOs. All three groups of HMOs consist of a lactose backbone that can be further elongated into complex structures [4]. In contrast to the wide variety of oligosaccharides in breast milk, infant formulas nowadays often contains lactose- (e.g. galactooligosaccharides) or plant-derived (e.g. fructooligosaccharides) oligosaccharides, that are best known for their prebiotic effect to mimic the functionality of HMOs in breast milk [8]. In addition to the prebiotic effects of HMOs and oligosaccharides used in infant formulas, milk oligosaccharides may have direct effects on the immune system [9–16]. However, breast milk contains over 140 different HMOs and the immunomodulatory potential of only a few has been investigated to date, and detailed mechanistic evidence is currently lacking.

Dendritic cells (DC) are unique in their ability to induce antigen-specific T cell responses. Monocytes differentiate into monocyte-derived DC in the presence of inflammatory cytokines. Recent evidence suggests that our mucosal surface is in a state of “primed homeostasis”, in which monocytes are recruited to the lamina propria [17]. Breast milk components such as TGFβ and the active form of vitamin D3 (i.e. 1α,25-dihydroxyvitamin D_3_) promote the differentiation of these recruited monocytes into tolerogenic DC [18,19]. Such tolerogenic monocyte-derived phagocytes are present in the GI tract and are essential to maintain oral tolerance in the lamina propria [20,21]. Additionally, components in breast milk may dampen inflammatory responses induced by immature DC upon activation by pathogens (i.e. maturation). As such, a complete pool of HMOs was shown to induce differentiation of bone marrow-derived cells into tolerogenic DC [22]. Immune regulation by such breast milk components may contribute to immune homeostasis.

To date no direct immunomodulatory effects of HMOs on human dendritic cell differentiation and maturation have been reported. We aimed to address this question by studying the effects of HMOs on DC differentiation and maturation. 6’sialyllactose (6’SL) and 2’fucosyllactose (2’FL) were used as representatives of acidic and neutral fucosylated HMOs and galacto-oligosaccharides (GOS) as neutral non-fucosylated oligosaccharides, which were compared to the known tolerogenic effects of TGFβ and 1α,25-dihydroxyvitamin D_3_ (vitD3) that were included as positive controls_._ First, the effect of 6’SL, 2’FL and GOS was investigated on differentiation by culturing monocytes with IL-4 and GM-CSF in the presence or absence of these oligosaccharides. Second, differentiated moDC, vitD3DC and TGFβDC were assessed for their expression of HMO-recognizing receptors. These DC types were used to study the effect of 6’SL, 2’FL and GOS on LPS-induced maturation.

## Material and methods

### Isolation and culturing of monocyte-derived DC types

Monocytes were isolated from PBMCs from healthy anonymous donors (Sanquin blood bank, Nijmegen) as described earlier [23]. Immature moDCs were generated by differentiating monocytes for 6 days in the presence of 20 ng/ml IL-4 (Peptrotech; 200–04) and GM-CSF (Peprotech; 300–03) in the presence or absence of TGFβ (3 ng/ml TGFβ1 + 10ng/ml TGFβ2, Peprotech; 100-21/100-35), 10nM VitD3 (Sigma-Aldrich, D1530), 0.5 mg/ml 2’FL (Inalco S.p.A.) or GOS (FrieslandCampina), or 0.375 mg/ml 6’SL (Carbosynth, OS04398). These immature moDC, TGFβDC or VitD3DC were stimulated (i.e. maturated) with 1 μg/ml *E*.*coli* OIII:B4 LPS (Invivogen; tlrl-3pelps) with or without 0.5 mg/ml GOS, 2’FL, or 0.375 mg/ml 6’SL for 48 hours.

### LPS detection

6’SL, 2’FL and GOS were tested for LPS contamination by a recombinant factor C LAL assay that was performed according to the manufacturers recommendations (EndoZyme recombinant factor C assay, Hyglos; 609050). Endotoxin levels of <0.05 EU/mg were found in GOS and 2’FL preparations. 6’SL contained trace amounts of LPS; 0.12 EU/mg.

### Isolation and staining of moDCs

DCs were put on ice for 30 minutes under constant shaking. The collected cells were stained with a mixture of conjugated monoclonal antibodies or matched isotype controls (S1 Table). Cell viability was analysed by staining the cells with DRAQ7 (Abcam; ab109202) or fixable viability dye ef506 (eBiosciences, 65-0866-14). Cells were acquired on a BD FACS Canto II (BD Biosciences) and analysed using the FlowJo software V10

### Quantification of cytokine levels in supernatants

Levels of IL-8 IL-6, IL-10, TNF-α and IL-12p70 were measured in the supernatants using a cytometric bead array technique (BD Biosciences). Individual flex-sets for IL-8 (558277), IL-6 (558276), TNF-α (560112), IL-10 (558274) or IL-12p70 (558283) were run according to the manufacturer’s instructions.

### Statistics

The raw data was tested for normality with a Kolmogorov-Smirov test. Data with unequal distributions were transformed using the logarithm or square root of the raw data. A linear mixed model with a LSD test was used on the raw or transformed data to compare the various treatment groups to the control (moDC) as a reference group unless stated otherwise. Significant differences were indicated by: *** = P< 0.001, ** = P< 0.01 and * = P< 0.05. All values are represented as mean +/- SEM. IBM SPSS Statistics V23.0 was used for the statistical analysis.

## Results

**Fig 1:**
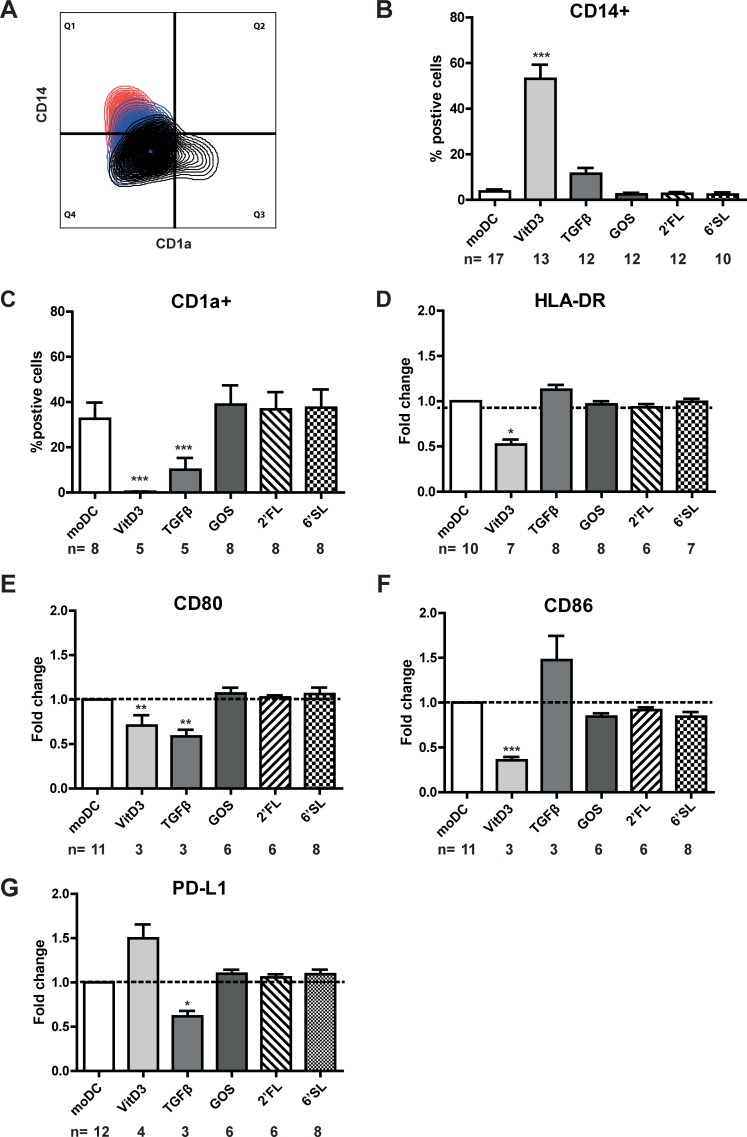
TGFβ and VitD3 induce phenotypic distinct DCs. CD14+ monocytes were cultured in the presence of IL-4 and GM-CSF for six days in the presence or absence of breast milk components. Surface marker expression was measured by flow cytometry. A) A multi-colour overlay of CD14 expression versus CD1a expression on moDC (black), TGFβDC (blue) or VitD3 (red) of one representative donor is shown. The percentage of B) CD14+ and C) CD1a+ DC and relative surface marker expression of D) HLA-DR, E) CD80, F) CD86 or G) PD-L1 on immature DC differentiated in the presence of TGFβ, VitD3, 6’SL, 2’FL or GOS was shown. Relative fold change was calculated by dividing the MFI (median fluorescence intensity) of DC differentiated in the presence of a breast milk component/MFI of moDC of each respective donor.

To investigate the effect of the breast milk components on DC differentiation, monocytes were differentiated into immature monocyte-derived DC (iDC) by culturing the cells for six days with IL-4 and GM-CSF in the presence or absence of individual breast milk component. 6’SL, 2’FL or GOS did not alter the expression of any of the measured surface markers on immature DC (Fig 1). Monocytes differentiated in the presence of VitD3 remained CD14+ and did not gain CD1a expression on their surface, showing that VitD3 was capable of halting DC differentiation (Fig 1A). Similarly, significantly less DC that were differentiated in the presence of TGFβ showed CD1a expression compared to moDC (Fig 1A). In contrast, 6’SL, 2’FL or GOS did not alter CD14 and CD1a expression (Fig 1B and 1C). Although fewer TGFβDC and VitD3DC showed to be CD1a+ compared to moDC, both DC types showed a different expression profile of activation markers. VitD3DC showed significantly lower HLA-DR (Fig 1D), CD80 (Fig 1E), CD86 expression (Fig 1F) and higher PD-L1 (Fig 1G) compared to moDCs. In contrast, TGFβDC expressed significantly lower levels of CD80 (Fig 1E) and PD-L1 (Fig 1G) on their surface compared to moDC.

**Fig 2:**
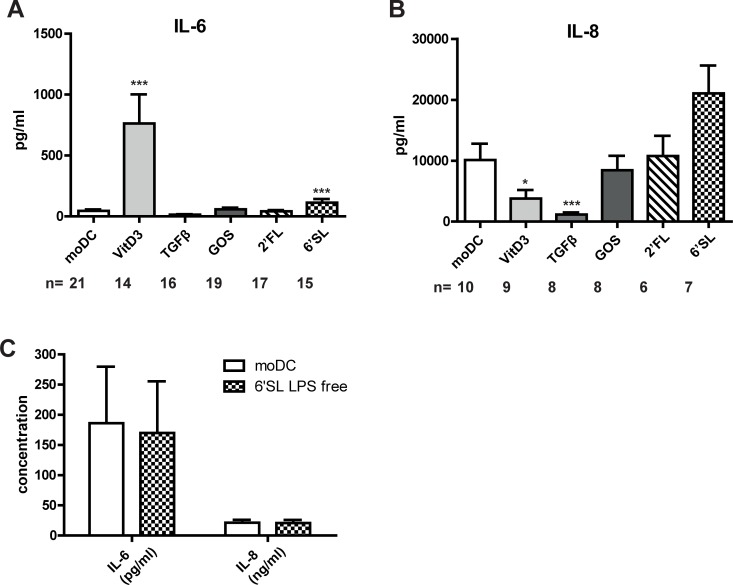
TGFβ and VitD3 differentially modulate the production of IL-6 and IL-8 during differentiation. 6’SL was treated with an optimized Triton X-114 method to remove LPS traces. Immature DC were cultured for six days in the presence or absence of VitD3, TGFβ, 6’SL, LPS-free 6’SL, 2’FL or GOS. A,C) IL-6 and B,C) IL-8 were measured in the supernatant by CBA.

To investigate whether breast milk components induced the production of cytokines during differentiation, we measured the cytokines in the supernatants that were produced during the differentiation period. Intriguingly, VitD3 induced the production of IL-6 (Fig 2A) and reduced the production of IL-8 (Fig 2B) during DC differentiation. TGFβ reduced the production of IL-8 and IL-6 (P = 0.08) during DC differentiation compared to moDC. IL-10 and TNF production was below the detection limit of the assay (<10 pg/ml). 6’SL induced an approximate two-fold increase in IL-6 production compared to moDC (Fig 2A) and a trend (P = 0.078) towards higher IL-8 levels (Fig 2B). However, since we measured trace amounts of 0.12EU/mg of LPS in this sample, we applied an optimized Triton X-114 method to remove LPS traces [24]. Triton X-114 treated 6’SL showed a similar production of IL-6 and IL-8, showing that the two-fold increase in IL-6 was caused by LPS traces (Fig 2C).

**Fig 3:**
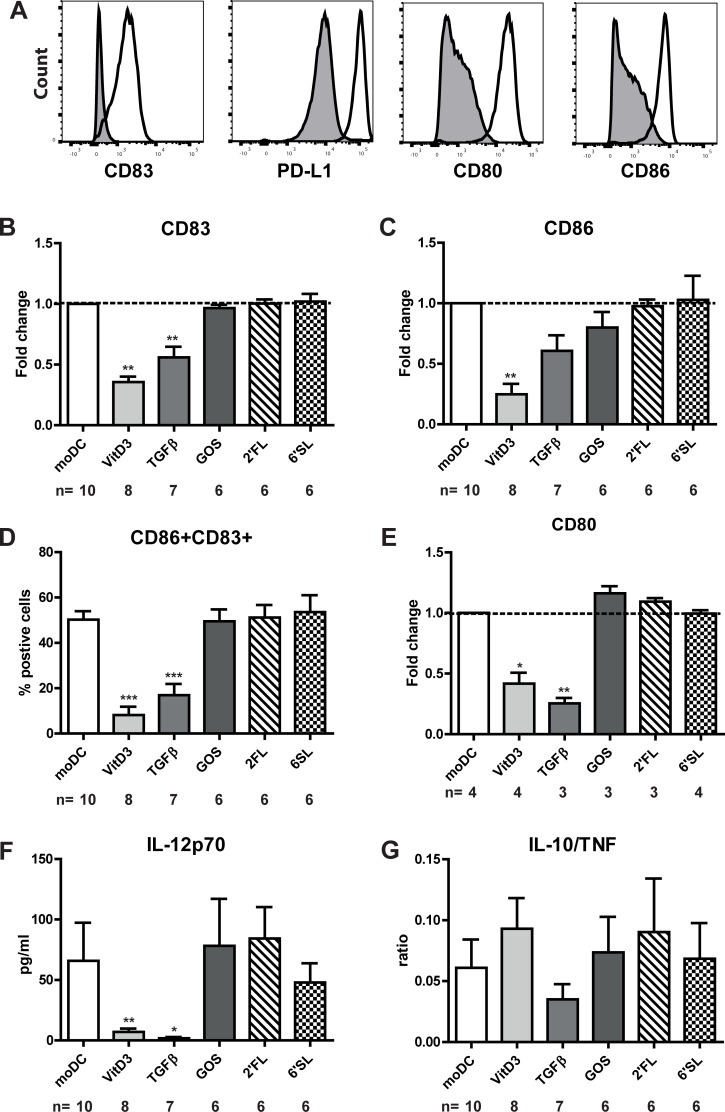
TGFβDC and VitD3DC induce tolerogenic DC. Immature DC were stimulated with LPS for 48 hours. A) The expression of CD83, PD-L1, CD80 and CD86 on immature DC (shaded histograms) or mature DC (open histograms) of one donor was shown. The relative surface marker expression of B) CD83, C) CD86 and E) CD80 are shown. Relative fold change was calculated by dividing the MFI (median fluorescence intensity) of DC differentiated in the presence of a breast milk component/MFI of moDC of each respective donor. D) Percentage of CD86+CD83+ DCs. F) IL-12p70 and IL-10 and TNF were measured in the supernatant by CBA. G) The IL-10/TNF ratio is shown for the different mature DC that were differentiated in the presence of different breast milk components.

After the differentiation period of six days, we assessed whether the DC types that were cultured in the presence of 6’SL, 2’FL, GOS, VitD3 or TGFβ were functionally different by stimulating these immature DC for 48 hours with LPS. moDC upregulated the expression of CD83, PD-L1, CD80 and CD86 upon LPS-induced maturation (Fig 3A). Immature dendritic cells that were generated in the presence of 6’SL, 2’FL or GOS did not show any phenotypic changes (Fig 3B–3E) or cytokine production (3F and 3G) upon maturation compared to moDC. VitD3DC and TGFβDC showed to be hyporesponsive towards LPS as shown by a semi-mature phenotype (i.e. reduced expression of both CD83 and CD86) (Fig 3B–3D) and lower expression of CD80 (Fig 3E), HLA-DR and PD-L1 (S1A and S1B Fig). In addition, VitD3DC and TGFβDC produced lower amounts of IL-12p70 after LPS stimulation compared to moDC (Fig 3F). However, the ratio of IL-10/TNF and quantitative IL-10 and TNF levels (S1C and S1D Fig) was not significant different compared to conventional DCs (Fig 3G). Thus we showed 6’SL, 2’FL and GOS, in contrast to vitD3 and TGFβ, did not induce differentiation of monocytes into tolerogenic DC.

**Fig 4:**
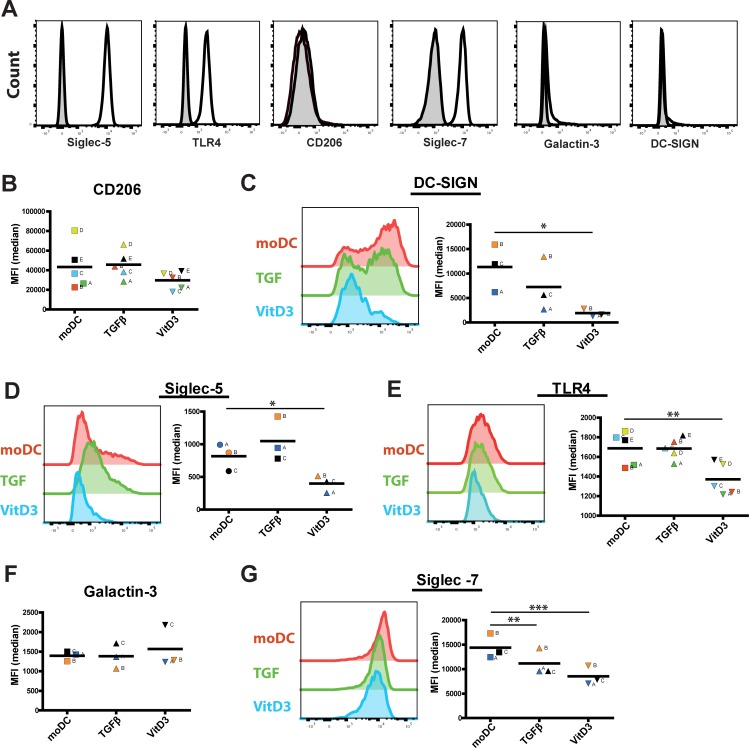
moDC, TGFβDC and VitD3DC express different levels of HMO-recognizing receptors. The expression of several receptors that are shown in literature to recognize HMOs were measured on A) monocytes and B-G) moDC, TGFβDC and vitD3DC. The expression of Siglec-5, Siglec-7, TLR4, CD206, Galactin-3 and DC-SIGN (open histograms) and their matching isotype control (shaded histograms) were measured on monocytes. The expression of B) CD206, C) DC-SIGN, D) Siglec-5, E) TLR4, F) Galactin-3 and G) Siglec-7 was shown by scatter plots (n = 3–5) and histogram of one representative donor. Normal distribution was assumed due to the low sample size. Significance was tested by a repeated measures ANOVA with a Tukey’s multiple comparison post-hoc test.

Next, we investigated the expression of HMO-recognizing receptors on moDC, TGFβDC and VitD3DC to assess their potential responsiveness towards 6’SL, 2’FL and GOS. Moreover, we investigated the expression of Siglec-5 and Siglec-7 as potential receptors for 6’SL. DC-SIGN and CD206 were included as potential receptors of 2’FL and galactin-3 as putative receptor for GOS. We also measured the expression of TLR4 since we used LPS to mature DC. Monocytes expressed high levels of Siglec-5, Siglec-7, and TLR4, no expression of DC-SIGN and CD206 and low expression of Galactin-3 (Fig 4A). The expression of CD206 (Fig 4B) and DC-SIGN (Fig 4C) was high after differentiation of the monocytes into moDC. In contrast, the expression of Siglec-5 was markedly lower after differentiation. Interestingly, VitD3DC showed significantly lower expression of Siglec-5, Siglec-7, DC-SIGN and TLR4 compared to moDC (Fig 4D–4F). TGFβDC of all three tested donors showed a lower expression of DC-SIGN (Fig 4C) and significant lower expression of Siglec-7 (Fig 4F) compared to moDC. This data thus showed that moDC, TGFβDC and vitD3DC express different levels of receptors that are known to recognize HMOs. 6’SL, 2’FL or GOS may therefore differentially modulate moDC, TGFβDC and vitD3DC.

**Fig 5:**
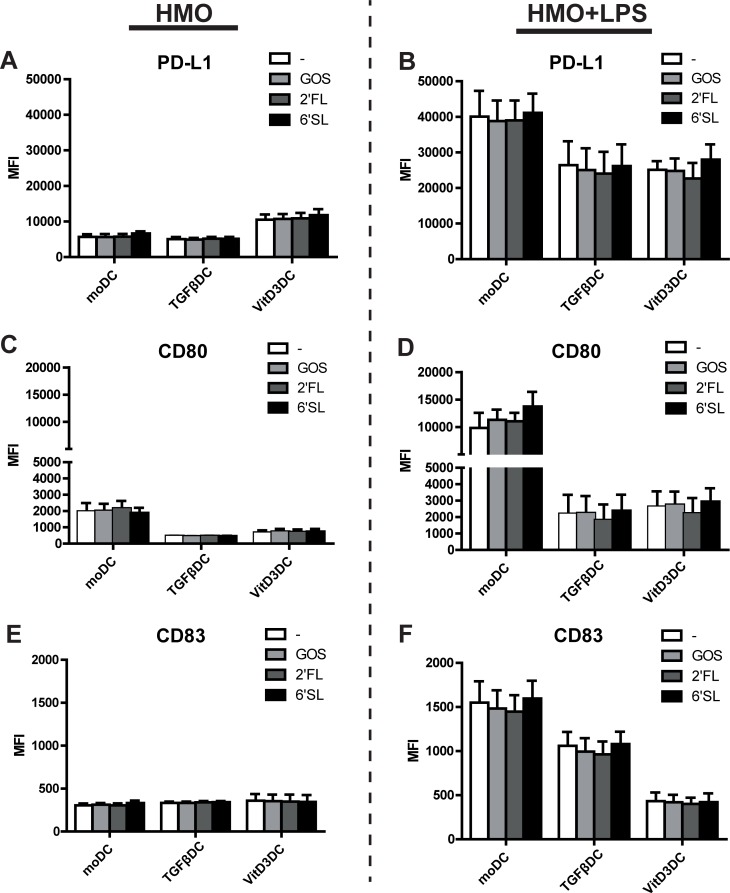
HMOs do not impact maturation of different in vitro generated DC. Immature moDC, TGFβDC and VitD3DC were stimulated with LPS with or without 6’SL, 2’FL or GOS for 48 hours. The effect of 6’SL, 2’FL and GOS was measured in the absence (A, C, F) or presence of LPS (B, D,F). The surface marker expression of A, B) PD-L1 and C, D) CD80 and E, F) CD83 is shown as MFI (median fluorescence intensity) (two independent experiments, six donors).

After having shown that moDC, TGFβDC or vitD3DC express different levels of TLR4 and HMO-recognizing receptors, we used these DC types to investigate the effect of 6’SL, 2’FL or GOS on LPS-induced maturation. While we observed differences in receptor expression, none of the oligosaccharides tested affected the phenotype of TGFβDC, VitD3DC or moDCs in the absence or presence of LPS compared to the respective DC type that was matured in the absence of HMOs (Fig 5). In line with these findings, 6’SL, 2’FL or GOS did not modulate the production of IL-6, IL-8, IL-10, IL-12p70 or TNF production by moDC, TGFβDC or VitD3DC in the presence or absence of LPS (S2 Fig). VitD3DCs showed a higher expression of PD-L1 compared to TGFβDC or moDC (Fig 5A), which was also seen before on day 6 of culture (Fig 1G). As shown in Fig 3 and S1 Fig, LPS stimulated VitD3DC and TGFβDC showed a lower expression of PD-L1, CD83 and CD80 compared to moDC (Fig 5C and 5D).

## Discussion

In this study we investigated the effect of 6’SL, 2’FL and GOS and the well-studied factors TGFβ and VitD3 on DC differentiation. None of the oligosaccharides tested affected DC differentiation. VitD3 and TGFβ induced phenotypical distinct immature DCs. These immature DC produced different levels of cytokines during differentiation compared to moDC. As shown by others, DCs differentiated in the presence of VitD3 or TGFβ showed a tolerogenic phenotype; showing hyporesponsiveness towards LPS and abrogated IL-12p70 production [19,25–27]. We showed that these moDC, TGFβDC and VitD3DC differentially express HMO-binding receptors and TLR4. However, 6’SL, 2’FL and GOS did not modulate LPS-induced maturation on any of these DC types.

The GI tract is constantly exposed to commensal microorganisms and their metabolites. As a result, monocytes are constantly recruited to the GI tract [17]. These monocytes differentiate into macrophages or dendritic cells in the presence of cytokines. Environmental factors (e.g. diet) can alter the differentiation of these monocytes into functionally distinct cells [18,19]. Oligosaccharides are largely fermented in the colon and a small fraction becomes systemically available [5,6]. In bone marrow-derived DC cultures, a complete fraction of HMOs was shown to induce regulatory responses [22]. It is therefore of interest to study the direct immunomodulatory effect of individual HMOs on monocytes and DC. The data presented here show that in contrast to TGFβ and vitD3, 6’SL, 2’FL and GOS have no immunomodulatory effect on the differentiation of monocytes into DC. Next, we used these immature moDC, TGFβDC and vitD3DC to measure the surface marker expression of HMO-recognizing receptors and subsequently test their responsiveness towards 6’SL, 2’FL or GOS. Receptors that are postulated to bind HMOs are CD206 and DC-SIGN, which are shown to bind 2’FL [28], while sialic-acid-binding immunoglobulin-like lectins (Siglecs) are known to bind sialic acid containing HMOs such as 6’SL. Siglecs contain intracellular tyrosin-based inhibitory (ITIM) motifs that dampen immune responses [29]. In line with literature, we showed that human monocytes express Siglec-5 and Siglec-7 [30]. We showed that the expression of Siglec-5 and -7 was markedly downregulated on TGFβDC and vitD3DC compared to moDC. Nevertheless, we observed no difference in the immunomodulatory effect of 6’SL on both DC types, indicating that 6’SL does not induce DC functioning via Siglec-7. This could be explained by the low binding affinity of 6’SL to Siglec-7 [29]. Similarly, activation of CD206, DC-SIGN and galactin-3 may result in dampening of inflammation [28,31–34]. GOS may bind galactin-3—a β-galactoside-binding lectin that is expressed on monocytes [31]. Although galactin-3 signalling has been shown to induce differentiation of monocytes into anti-inflammatory alternative activated macrophages [32], we did not show any phenotypical changes if GOS was added during DC differentiation. Thus, we showed that although TGFβDC and vitD3DC express different levels of receptors that may recognize HMOs, 6’SL, 2’FL or GOS did not modulate DC maturation.

In contrast, others do show immunomodulation by HMOs *in vitro* [9,12,13,22]. For instance, the acidic HMO fraction of breast milk was—in contrast to the neutral HMO fraction—shown to induce the frequency IFNγ+ T cells and CD4+CD25^+^ regulatory T cells in a human mononuclear cord blood cell culture [13]. This immunoregulatory effect of the acid fraction was sufficient to restore the Th1/Th2 cytokine balance in PBMCs of peanut allergic patients stimulated with the major allergen Ara h1 [14]. In mouse pups, the acidic trisaccharide 3’SL was shown to promote colitis by inducing inflammation in a TLR4 mediated manner [12]. The prebiotic oligosaccharides scGOS/lcFOS mixture, FOS and inulin were also shown to exert immunomodulation on monocytes via TLR4 [10,15]. Here we show that 6’SL, which is abundantly present in breast- and cow’s milk [35], induces the production of IL-6 and IL-8 during DC differentiation and did not alter the phenotype. However, after applying an optimized Triton X-114 method [24], we showed that this twofold increase was caused by trace amounts of LPS (0.12 EU/mg). We have previously shown that a 100-fold higher contamination of LPS in commercial 3’SL results in rapid IL-6 and IL-8 production and induces differentiation into tolerogenic DC [23]. Since LPS is an important contaminant that signals via TLR4, caution must be applied interpreting direct immunomodulation of HMOs via TLR4. Even though we could not demonstrate effects of 6’SL, 2’FL or GOS on monocytes and dendritic cells, it is possible that other HMOs or other prebiotic oligosaccharides can have immunomodulatory effects on monocytes and dendritic cells, since breast milk contains at least 140 different HMOs. Additionally, HMOs have been shown to have an important immunomodulatory role indirectly by altering the microbiota composition and enhancing the production of short chain fatty acids (SCFA). These SCFA produced by the microbiota are known to exert immunosuppressive effects on moDC [36].

VitD3 and TGFβ altered the differentiation into phenotypical distinct immature DCs. Both of these immature VitD3DC and TGFβDC types are hyporesponsive towards LPS. The notion that VitD3 and TGFβ induce phenotypical different DC types, which produce a very different cytokine profiles during differentiation, indicates that different pathways are responsible for the differentiation into regulatory DCs. This hypothesis is in line with literature showing distinct pathways involved in NF-κB regulation by VitD3 and TGFβ on human monocyte-derived DC [27]. Although TGFβ and VitD3 may act via different mechanisms, we show in this study that both are capable of halting DC differentiation. One possible mechanism by which VitD3 inhibits DC differentiation is by the inhibition of GM-CSF signaling via upregulation of SOCS1 [37,38], which could halt DC differentiation. The mechanism by which TGFβ influences DC development is not well established. Since the SMAD-induced pathways was shown to be active in moDC [27], TGFβ may halt DC differentiation via this pathway. Interestingly, we show here that CD1a is expressed on fewer TGFβDCs compared to moDCs. CD1a+ DCs do in contrast to DCs produce IL-12p70 upon stimulation [39], which we also observed for VitD3DCs and TGFβDCs. Additionally, we showed that VitD3 and not TGFβ triggers the production of IL-6 during DC differentiation. IL-6 in turn can induce STAT3 activation *in vitro* and *in vivo* and halts DC differentiation [40,41]. STAT3 binds the PD-L1 promotor directly and regulates its expression [42]. Indeed, we showed that vitD3 triggers IL-6 production and that PD-L1 expression is higher on immature VitD3DCs compared to TGFDCs or moDC. Thus, VitD3DCs and TGFβDCs are less responsive to LPS (i.e. tolerogenic DC), as observed by their semi-mature phenotype and lower cytokine production, which is line with literature [18,19,25,43].

## Conclusions

The GI tract is constantly repopulated by monocyte derived DC and macrophages due to the exposure of microbes and their metabolites. Breast milk contains components such as VitD3 and TGFβ that can alter the differentiation of monocytes into tolerogenic DCs. Even though oligosaccharides are thought to play an important role in immune development in early life, the HMOs and GOS tested here do not alter DC differentiation or maturation of *in vitro* differentiated mucosal DC types. Further unravelling the impact of these and other breast milk components on immune homeostasis will improve our understanding of how breastfeeding promotes immune homeostasis and development. This knowledge can be applied to develop new strategies to protect infants against infections and allergies.

## Supporting information

S1 Fig TGFβ and VitD3 induce tolerogenic DC. Immature DC differentiated in presence of 6’SL, 2’FL, GOS, TGFβ or VitD3 were stimulated with LPS for 48 hours. The relative surface marker expression of A) HLA-DR and B) PD-L1 are shown. Relative fold change was calculated by dividing the MFI (median fluorescence intensity) of DC differentiated in the presence of a breast milk component/MFI of moDC of each respective donor. C) TNF and D) IL-10 were measured in the supernatant by CBA.
(EPS)

S2 Fig HMOs do not impact maturation of different in vitro generated DC. Immature moDC, TGFβDC and VitD3DC were stimulated with LPS with or without 6’SL, 2’FL or GOS for 48 hours. The effect of 6’SL, 2’FL and GOS on cytokine production was measured in the absence (A and C) or presence of LPS (B, D-G). A, B) IL-8, C, D) IL-6, E) IL-10, F) IL-12p70 and G) TNF were measured by CBA.
(EPS)

S1 Table Conjugated antibodies used for flow cytometry. (DOCX)
