# Supplementary material for: The oligosaccharides 6’-sialyllactose, 2’-fucosyllactose or galactooligosaccharides do not directly modulate human dendritic cell differentiation or maturation
Source: PLoS One. 2018 Jul 10;13(7):e0200356. doi: 10.1371/journal.pone.0200356 (PMC6039038; doi:10.1371/journal.pone.0200356)
Supplement: S1 Table — (DOCX) [file pone.0200356.s003.docx]

| **Target** | **Fluorochrome** | **Company** | **Cat.nr. Ab** | **Cat. nr. Isotype control** |
| --- | --- | --- | --- | --- |
| CD14 | FITC | BD Biosciences | 555397 | 555573 |
| CD83 | FITC | BD Biosciences | 556910 | 555573 |
| CD1a | PerCP-Cy-5 | Biolegend | 300130 | 400149 |
| CD86 | V450 | BD Biosciences | 560357 | 560373 |
| HLA-DR | APCef780 | eBiosciences | 47-9956-42 | 47-4732-80 |
| CD80 | PE-Cy-5 | BD Biosciences | 559370 | 555576 |
| PD-L1 | PE-Cy7 | BD Biosciences | 558017 | 557872 |
| Galactin-3 | Alexa Fluor 488 | Biolegend | 125410 | 553929 |
| Siglec-5 | PE | Biolegend | 352003 | 555573 |
| Siglec-7 | APC | Biolegend | 339205 | 17-4714-81 |
| DC-SIGN | BV421 | BD Biosciences | 566278 | 562438 |
| CD206 | PE-Cy7 | eBiosciences | 25-2069-42 | 552868 |
| TLR4 | BV421 | BD Biosciences | 5167523 | 562438 |

**S1 Table. Conjugated antibodies used for flow cytometry**
